# Air Pollution and Emergency Department Visits for Mental Disorders among Youth

**DOI:** 10.3390/ijerph17124190

**Published:** 2020-06-12

**Authors:** Mieczysław Szyszkowicz, Roger Zemek, Ian Colman, William Gardner, Termeh Kousha, Marc Smith-Doiron

**Affiliations:** 1 Environmental Health Science and Research Bureau, Health Canada, Ottawa, ON K1A 0K9, Canada; marc.smith-doiron@canada.ca; 2 Department of Pediatrics and Emergency Medicine, Children’s Hospital of Eastern Ontario, University of Ottawa, Ottawa, ON K1N 6N5, Canada; RZemek@cheo.on.ca; 3 School of Epidemiology and Public Health, University of Ottawa, Ottawa, ON K1N 6N5, Canada; icolman@uottawa.ca; 4 School of Epidemiology and Public Health, Faculty of Medicine, University of Ottawa, Ottawa, ON K1N 6N5, Canada; WGardner@cheo.on.ca; 5 Department of Mathematics and Statistics, University of Ottawa, Ottawa, ON K1N 6N5, Canada; tkousha@uottawa.ca

**Keywords:** ambient air pollution, AQHI, concentration, exposure, mental health, paediatric, adolescent, young

## Abstract

Although exposure to ambient air pollution has been linked to mental health problems, little is known about its potential effects on youth. This study investigates the association between short-term exposure to air pollutants and emergency department (ED) visits for mental health disorders. The National Ambulatory Care Reporting System database was used to retrieve ED visits for young individuals aged 8–24 years in Toronto, Canada. Daily average concentrations of nitrogen dioxide (NO_2_), fine particulate matter (PM_2.5_), and daily maximum 8 h ozone (O_3_) were calculated using measurement data from seven fixed stations. A case-crossover (CC) design was implemented to estimate the associations between ED visits and air pollution concentrations. Mental health ED visits were identified using International Classification of Diseases 10th Revision (ICD-10) codes, with seven categories considered. Models incorporating air pollutants and ambient temperature (with lags of 0–5 days) using a time-stratified CC technique were applied. Multivariable regression was performed by sex, three age groups, and seven types of mental health disorders to calculate relative risk (RR). The RRs were reported for one interquartile range (IQR) change in the air pollutant concentrations. Between April 2004 and December 2015 (4292 days), there were 83,985 ED visits for mental-health related problems in the target population. Several exposures to air pollutants were shown to have associations with ED visits for mental health including same day exposure to fine particulate matter (IQR = 6.03 μg/m^3^, RR = 1.01 (95% confidence interval: 1.00–1.02), RR = 1.02 (1.00–1.03)) for all and female-only patients, respectively. One-day lagged exposure was also associated with ED visits for PM_2.5_ (RR = 1.02 (1.01–1.03)), for nitrogen dioxide (IQR = 9.1 ppb, RR = 1.02 (1.00–1.04)), and ozone (IQR = 16.0 ppb, RR = 1.06 (1.01–1.10)) for males. In this study, urban air pollution concentration—mainly fine particulate matter and nitrogen dioxide—is associated with an increased risk for ED visits for adolescents and young adults with diagnosed mental health disorders.

## 1. Introduction

Urban air pollution concentrations are usually represented by three ambient air pollutants: fine particulate matter, ozone, and nitrogen dioxide. The modes of entrance of air pollutants into the human body are limited, but the resultant physiological responses can present a large spectrum of pathologies. Air pollution exposure may trigger unexpected and unpredictable conditions, such as inflammation. Inflammatory processes may play a crucial role in the etiology of many psychiatric disorders [1,2,3,4]. Ambient air pollution is a serious environmental issue which seriously affects various aspects of human health [5,6]. It is listed among numerous other factors proposed in the etiology of youth mental disorders [7]. Associations have been reported between air pollution and depression [8,9,10,11,12,13,14,15], anxiety [16], suicide [17,18,19,20,21,22,23], and drug abuse [24]; however, the majority of this research has been focused on adult populations. This implies that a connection between air pollution and mental health disorders is plausible [25]. Experimental research in mice [26,27] and post-mortem studies on humans [28] have indicated that air pollutants—mainly fine and ultrafine particulate matter—are capable of reaching the brain.

Mental health conditions among children and adolescents are highly prevalent. It is estimated that 10–20% of young people are affected by some type of mental disorder [29,30]. Another study stated that an estimated 20% of Canadians will experience a mental disorder during their lifetime, with the onset generally occurring at a young age [31].

The existing literature indicates an association between high air pollution in cities and an elevated presence of child and adolescent mental conditions [32,33,34]. One of the environmental factors which have been identified as a cause of mental disorder is poor air quality in urban environments [35]. Children and youth are most vulnerable to air pollution and may be especially susceptible to neurological impacts as their brains are still developing [36].

In this study, we considered the emergency department (ED) visits diagnosed and recorded as mental health conditions among persons between the ages of 8 and 24 years old. We considered the following mental disorder types: organic, substance-related, schizophrenic and psychotic, mood, anxiety, personality, and other disorders.

The main hypothesis in this work is that exposure to ambient air pollution is associated with an elevated risk of mental health episodes that result in ED visits among young individuals.

## 2. Materials and Methods

### 2.1. Study Population

The population studied were individuals between 8 and 24 years of age visiting EDs in Toronto, ON, Canada. Data on ED visits were retrieved from the National Ambulatory Care Reporting System database (NACRS) [37]. The NACRS is a reporting system for ED cases in Canada. The system represents more than 97% of the ED visits in the province of Ontario. The NACRS database is accessible through Health Canada. SAS Guide 7.1 software (SAS Institute, Cary, NC, USA) was applied to retrieve the corresponding values related to ED visits. The records contain patient basic information (sex, age, date of ED visit).

The considered area of the study is determined by the Census Division (CD) of Toronto. Its land area is 630.2 square kilometers. The population density of this region is 4334.4 people per square kilometer. In the year 2016, the enumerated population of the CD of Toronto was 2,731,571 people, which is an increase of 6.2% from the year 2011. ED visits by location within the city of Toronto were classified using the first three alphanumeric characters of available postal codes for each patient’s home address.

### 2.2. Environmental Data

We considered the following three ambient air pollutants: nitrogen dioxide (NO_2_), daily maximum 8 h ozone (O_3_), and fine particulate matter (PM_2.5_), where the particles are no greater than 2.5 microns in diameter. The hourly air pollutant concentrations were recorded by ambient air quality monitoring stations in Toronto managed by the National Air Pollution Surveillance (NAPS) network in Canada [38]. Daily concentration levels were calculated as an average among the monitors. The NAPS stations and hospitals that were within the same area of study were selected. There were seven fixed air pollution monitoring stations in the metropolitan Toronto area.

In the constructed statistical models, daily average ambient temperature was included in the form of natural splines with three degrees of freedom. The temperature lagged by the same number of days as air pollutants, from 0 to 5 days.

### 2.3. Health Outcomes

ED visits for mental disorders were identified by applying the International Classification of Diseases 10th Revision (ICD-10) codes in Canada. Mental health conditions were grouped into seven types: organic disorders, substance-related disorders, schizophrenic and psychotic disorders, mood disorders, anxiety disorders, personality disorders, and other. In the Supplemental Materials, a list of all ICD-10 codes used is provided (available at https://github.com/szyszkowiczm/TorontoYoungMental). The data were grouped and analyzed by sex and age group. Mental health disorders are undercounted in ED records, largely because coders are only required to list a primary diagnosis [39]. The corresponding health data were available for the period between April 2004 and December 2015 (4292 days).

### 2.4. Statistical Methods

A summary of daily visits, as counts to the emergency department, were calculated and used in the statistical models. The analysis was performed for all patients, males, and females, and divided into three age groups [8–12], [13–18], and [19–24]. In addition, the calculations were done separately by the three age groups and separately for each of the seven types of mental disorders. 

This study examines the short-term effects of ambient air pollution on mental disorders using a case-crossover (CC) methodology [40]. A time-stratified technique was used to define the control periods [41]. In this approach, the same weekdays in a particular month were grouped and health risk was estimated by considering case and control exposures. In the standard CC method, conditional logistic regression is used to estimate the odds ratio (OR). In this paper, conditional Poisson models were applied as a flexible alternative approach to the case-crossover methodology [42,43,44,45,46]. In this technique, daily counts were considered rather than separate daily events. To this end, we created strata in which days were grouped in the nested hierarchical structure: days in weekdays, weekdays in months, and months in the corresponding years [42]. The conditional Poisson models were created with respect to the constructed strata. Using these models, the relative risks (RR) were estimated. The structure of the strata mimicked the time-stratified construction of case and control periods in the standard CC methodology. Within each stratum, the regression was performed with respect to the air pollution concentration levels; one stratum contained 4 or 5 days depending on the length of the month (28, 29, 30, or 31 days) and the day of week in a considered month (stratum = <year:month:day of week>).

The following statistical models were realized in the R computer language (R Core Team, 2019) using the gnm routine (generalized nonlinear model) to estimate the values of the concentration–response relationship coefficients (β):

model <− gnm(H ~ AP + ns(Temperature,3), data = dataIN, family = quasipoisson, eliminate = factor(stratum)),

where H is a response (daily counts of ED visits), AP is an exposure (air pollution concentration), and temperature is represented by natural splines with three degrees of freedom. The dataIN file contains the data with daily counts of ED visits (by the considered categories), daily concentration of air pollutants, and daily average of temperature. To model over-dispersed count data, the quasi-Poisson family is declared. In these models, time (one of the main factors of the study) was controlled by the constructed strata. 

For the purpose of sensitivity analysis, two approaches were used. The time period of the study was halved into two intervals, April 2004–December 2009 and January 2010–December 2015, and an identical statistical analysis was performed separately for both intervals. Another approach was to use an amalgamated mixture of air pollutants: Air quality in Canada is commonly quantified by the air quality health index (*AQHI*), the values for which are calculated according to the following formula:

$$AQHI=\frac{1000}{10.4}\left\{e^{0.000871*NO_{2}}+e^{0.000537*O_{3}}+e^{0.000487*PM_{2.5}}-3\right\}.$$

As the formula shows, three air pollutants are used to determine the values of the AQHI. The coefficients were estimated from a time-series study of air pollution and mortality from multiple Canadian cities [47]. Using the AQHI values allows the modeling of exposure to multiple air pollutants in common statistical models. In this approach, the associations of health conditions with three main urban air pollutants is assessed.

#### Ethics

The Health Canada Research Ethic Board determined that the study is Institutional Review Board (IRB) exempt, given that patient data were pre-existing and de-identified.

## 3. Results

Over the course of the study period, from 1 January 2004 to 31 December 2015, there were 83,985 ED visits by young individuals for mental health issues. Among these diagnosed visits, 41,176 (49%) were for males and 42,809 (51%) were for females. Table 1 summarizes the considered ED visits by type of health problems, sex, and age group.

The numerical results were obtained for various configurations of the considered health outcomes; for example, for all patients, all mood disorders, or male age group (ages 19–24) diagnosed with schizophrenic and psychotic disorders. As there are many considered configurations, the majority of the results are documented in the Supplementary Materials (Figures e1–e4 show the results by the categories of mental disease, Table e1 presents RRs and 95% CIs for two time periods; Table e2 presents RRs and 95% CIs for the AQHI; three data files with the corresponding numerical values generated by the all tested statistical models). Table 2 presents statistics on the daily averages of the considered environmental factors and ED visits.

**Figure 1:**
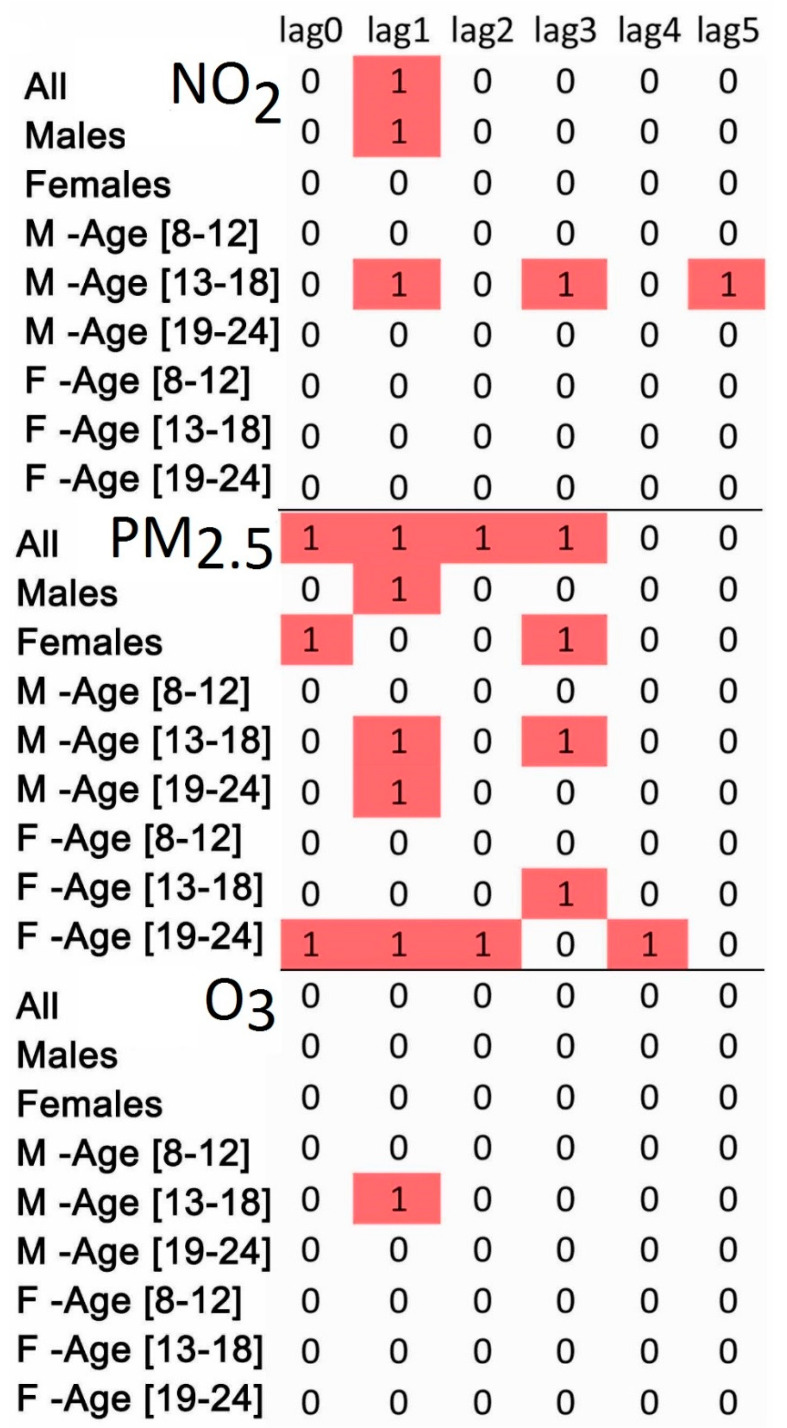
Qualitative representation of the associations by sex and age group for three air pollutants: red (1)—positive statistically significant, white (0)—neutral. F—female, M—male. Lags are in days. Toronto, Canada, 2004–2015.

Figure 1 shows qualitative results with three factors—age, sex, and pollutant—where the red color indicates that the results have a statistically significant positive association. The figure can be seen as a map to identify two kinds of associations: positive and null. For example, in the case of NO_2_, exposure at lag 0 has a cell value of 0 and that at lag 1 has a cell value of 1, which means that there is no association for lag 0 and a positive statistically significant association for lag 1. In the case of PM_2.5_, lags from 0 to 3 days have positive associations, as opposed to lags 4 and 5. In the Supplementary Materials, values of estimated slopes (Beta), their standard errors (SEBeta), P-values, and estimated RRs with their 95% confidence intervals (Cis) are listed in the paragraphs titled ResAgeGSex, ResAll, ResTypeAMF, where Res refers to results, AgeG refers to the age group, A refers to all patients, M refers to males, and F to females.

**Figure 2:**
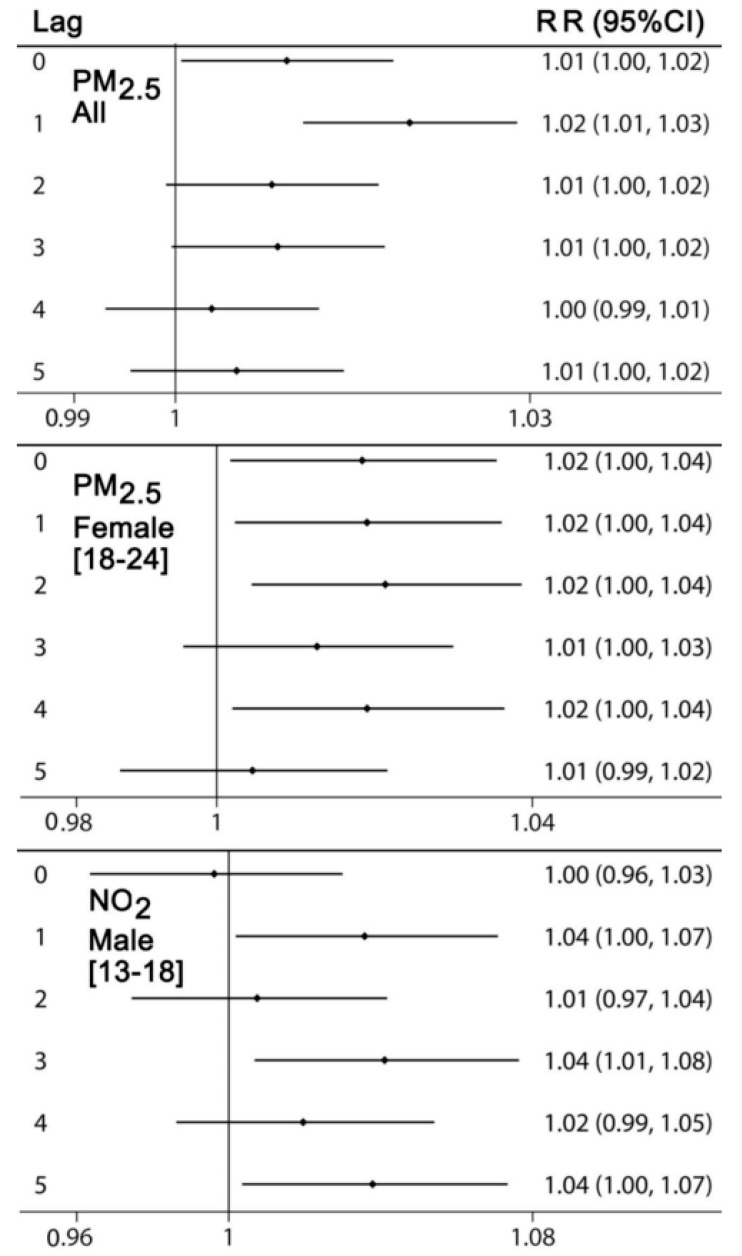
Relative risks (RR) and their 95% confidence intervals (CI) for fine particulate matter (PM_2.5_) (all patients and female individuals of age 19 to 24 years) and NO_2_ (male individuals of age 13 to 18 years). Lags are in days. Toronto, Canada, 2004–2015.

Figure 2 reports the estimated relative risk (RR) and 95% confidence intervals (CI) for an increase in air pollutant concentration levels measured by one interquartile range (IQR; nitrogen dioxide IQR = 9.1 ppb, and fine particulate matter IQR = 6.03 μg/m^3^).

**Figure 3:**
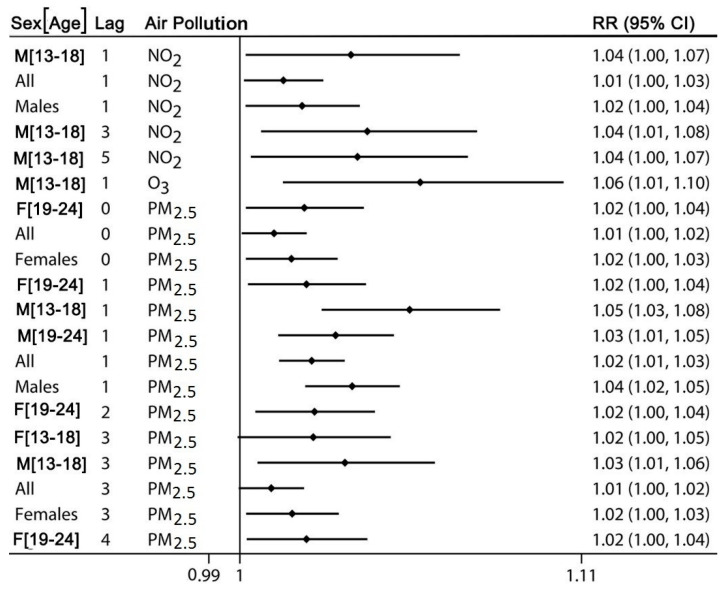
Relative risks (RR) and their 95% confidence intervals (CI) for positive statistically significant results. F—female, M—male. Lags are in days. Toronto, Canada, 2004–2015.

Figure 3 summarizes all positive statistically significant associations (ozone IQR = 16.0 ppb). The same representation convention is used in the Supplementary Materials.

The results obtained in the sensitivity analysis validate the associations between fine particulate matter lagged by 1 day and ED visits for all patients and males in the majority of cases. The results for all patients are as follows: for the first half of the study period, the RR is 1.0184 (1.0042–1.0328), and for the second half, the RR is 1.0245 (1.0082–1.0412). The results for males are RR values of 1.0406 (1.0203–1.0613) and 1.0259 (1.0022–1.0501), respectively (Table e1). In the Supplementary Materials (Table e1), the results verified for all positive statistically significant associations estimated for the whole time period are shown. All RRs are greater than one, although some of them are not statistically significant. This can be related to the statistical power, as the size of the data is around half that of the original.

The results for the AQHI support these associations for one day-delayed exposure over the whole period of the study. The estimations are as follows: for all patients, the RR is 1.0199 (1.0058–1.0342), while for males, the RR is 1.0305 (1.0102–1.0512), as shown in Table e2. The AQHI values depend on three major urban air pollutants.

In summary, these findings show that there are associations between fine particulate matter pollution and the number of ED visits for mental health disorders among young individuals. The results are positively statistically significant for “all patients” for lags of 0 to 3 days, and lags of 0 to 2 days for females older than 18. We observed these associations with an exposure lagged by 1 day. Previous-day exposure to PM_2.5_ shows a relationship with anxiety, mood, personality, and schizophrenic/psychotic disorders for all patients and male individuals. Nitrogen dioxide has associations with anxiety and mood disorder for males and females combined, as well as males only and females only.

## 4. Discussion

This study has shown that in Toronto—the largest and most populous Canadian city—there is an association between exposure to urban air pollutants and the number of ED visits for various mental disorders in children and young individuals. The results are presented for various subgroups by mental health category, age group, and sex. The majority of the results are provided in the Supplementary Materials. These values are suitable for further studies related to meta-analysis.

Fine particulate matter—the main component in ambient air pollution—had the most consistent statistically significant associations with mental health disorders. The chemical composition of particulate matter is also a significant factor, as it may by specific to the city of Toronto.

In a recent study related to fine particulate matter and psychotic episodes during adolescence [34], the estimated OR was 1.45 (95% CI: 1.11, 1.90). In the present study, the primary results (Figure 1) generated in the sensitivity analysis (Table e1) support the association of fine particulate matter (lagged by one day) with ED visits for mental disorders.

This research adds new evidence on how exposure to air pollutants affects our health, including mental health problems. Air pollution has been proven to cause stress hormone increases and to alter metabolic behavior [4], and has been shown to cross the blood–brain barrier [28]. Several studies have indicated associations between air pollutants and impaired cognitive function [48,49,50]. Chronic exposure to traffic-related air pollution (nitrogen dioxide and particulate matter) has been associated with decreased neurobehavioral function [51]. Animal studies also seem to support these associations, as mice who were exposed long-term to air pollution exhibited cognitive deficits and depression [26].

The obtained results are supported in other studies. Vert and colleagues [52] investigated depression in Barcelona and obtained significantly increasing results with a doubling odds ratio (OR = 2.00 (1.37, 2.93)) with a 10 μg/m^3^ NO_2_ increase. The authors also reported associations between higher concentration levels of air pollutants and psychiatric medication usage. A similar conclusion was obtained by a study of four Swedish counties [53]. It was found that children living in an area with higher levels of PM_2.5_ and NO_2_ have an elevated hazard ratio (HR) to having been dispensed psychiatric medication. The HR associated with a 10 μg/m^3^ increase in NO_2_ was estimated as 1.09 (95%CI: 1.06, 1.12). Some authors have identified higher concentration levels of ambient air pollutants in early childhood to be weakly correlated with psychotic disorders later in life [54,55].

There are limitations in the interpretation of the findings of this type of observational research. These include the adequacy of the statistical models used, measurement errors related to health condition diagnosis, and environmental variables. Another limitation is the assumption that each individual has the same exposure level, as the exposure here is represented as an average of concentration levels in a large urban area. Misclassification regarding the diagnosis of ED visits might also have confounded the results. Many individuals with mental disorders do not seek medical attention; however, this should introduce non-differential misclassification and bias our results towards the null.

There are limitations in the NACRS mental health codes used to identify health outcomes. Mental health disorders may not be reported if the primary reason for the visit was some other health issue. The formal mental health assessments are rarely conducted in ED settings, and most ED personnel do not have extensive mental health training.

In this type of study, causation cannot be confidently established, although it is possible to observe and accept a pattern of associations. In Canada, air pollution concentrations are relatively low when compared to other countries (Table 2). In the city of Toronto, vehicle traffic (including passenger vehicles) is a major contributor of the air pollutants examined in this study. Traffic intensity increases vehicle emissions and degrades ambient air quality, producing significant amounts of nitrogen oxides, carbon monoxide, and other pollution. Elevated noise is also characteristic of large agglomerations such as Toronto, which to some degree can confound the results in this study. While there is the possibility of unknown confounders, the case-crossover design should help mitigate this problem.

## 5. Conclusions

In this study, urban ambient air pollution is associated with an increase in the number of ED visits for mental health among children and young adults. The results are in line with those obtained in other centers. The estimated values of the associated risks are lower as, in Canada, the concentration levels of ambient air pollution are relatively low. Urban fine particulate matter may be very specific in terms of its components (such as metals, soot, crustal materials, organic matter, ammonium sulfate, and non-crustal trace elements) and vary by location.

## Figures and Tables

**Table 1:** Emergency department (ED) visits by type of health problem, sex, and age group. Toronto, Canada, 2004–2015.

Sex[Age]	Org	SubRel	SchPsy	Mood	Anx	Per	Oth	Total
M[8–12]	82	11	81	185	435	23	1110	1927
M[13–18]	186	3662	1540	2514	1774	178	2051	11,905
M[19–24]	219	8405	7107	4718	4777	444	1674	27,344
Male	487	12,078	8728	7417	6986	645	4835	41,176
F[8–12]	44	11	93	242	492	9	526	1417
F[13–18]	181	3460	1169	4835	3104	438	2722	15,909
F[19–24]	229	6019	3486	6597	6009	1067	2076	25,483
Female	454	9490	4748	11,674	9605	1514	5324	42,809
All	941	21,568	13,476	19,091	16,591	2159	10,159	83,985

Notes: M—male, F—female, [a–b]—age group. The abbreviations used are listed in the Supplementary Materials.

**Table 2:** Statistics of the daily values of the used environmental parameters. Toronto, Canada, 2004–2015. AQHI: air quality health index.

Parameters	Temp	O_3_	NO_2_	PM_2.5_	AQHI	ED Visit
Minimum	−22.2	3.3	4.0	0.1	1.0	2
Q1	1.7	24.0	12.0	3.8	2.4	15
Median	10.0	31.5	16.1	6.0	2.8	19
Mean	9.5	33.1	17.3	7.7	2.9	20
Q3	18.4	40.0	21.2	9.8	3.3	23
Maximum	31.2	85.9	62.3	44.8	7.1	60

Notes: Temp—temperature (in °C), Q1–25th percentile, Q3–75th percentile, O_3_ and NO_2_ in ppb, PM_2.5_ in μg/m^3^. ED Visit—all considered daily visits.

## Supplementary Materials

The numerical results from the performed statistical models are available online at https://github.com/szyszkowiczm/TorontoYoungMental.
